# Molecular modeling of major tobacco alkaloids in mainstream cigarette smoke

**DOI:** 10.1186/s13065-016-0189-5

**Published:** 2016-07-15

**Authors:** Caren Kurgat, Joshua Kibet, Peter Cheplogoi

**Affiliations:** Department of Chemistry, Egerton University, P.O Box 536, Egerton, 20115 Kenya

**Keywords:** Alkaloid, Bond dissociation energy, Toxicology, Density functional theory

## Abstract

**Background:**

Consensus of opinion in literature regarding tobacco research has shown that cigarette smoke can cause irreparable damage to the genetic material, cell injury, and general respiratory landscape. The alkaloid family of tobacco has been implicated is a series of ailments including addiction, mental illnesses, psychological disorders, and cancer. Accordingly, this contribution describes the mechanistic degradation of major tobacco alkaloids including the widely studied nicotine and two other alkaloids which have received little attention in literature. The principal focus is to understand their energetics, their environmental fate, and the formation of intermediates considered harmful to tobacco consumers.

**Method:**

The intermediate components believed to originate from tobacco alkaloids in mainstream cigarette smoke were determined using as gas-chromatography hyphenated to a mass spectrometer fitted with a mass selective detector (MSD) while the energetics of intermediates were conducted using the density functional theory framework (DFT/B3LYP) using the 6-31G basis set.

**Results:**

The density functional theory calculations conducted using B3LYP correlation function established that the scission of the phenyl C–C bond in nicotine and β-nicotyrine, and C–N phenyl bond in 3,5-dimethyl-1-phenylpyrazole were respectively 87.40, 118.24 and 121.38 kcal/mol. The major by-products from the thermal degradation of nicotine, β-nicotyrine and 3,5-dimethyl-1-phenylpyrazole during cigarette smoking are predicted theoretically to be pyridine, 3-methylpyridine, toluene, and benzene. This was found to be consistent with experimental data presented in this work.

**Conclusion:**

Clearly, the value of the bond dissociation energy was found to be dependent on the π–π interactions which plays a primary role in stabilizing the phenyl C–C in nicotine and β-nicotyrine and the phenyl C–N linkages in 3,5-dimethyl-1-phenylpyrazole. This investigation has elucidated the energetics for the formation of free radicals and intermediates considered detrimental to human health in cigarette smoking.

**Graphical abstract:**
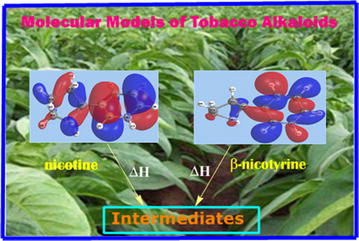
Some molecular alkaloids of tobacco the plant

**Electronic supplementary material:**

The online version of this article (doi:10.1186/s13065-016-0189-5) contains supplementary material, which is available to authorized users.

## Background

**Fig. 1:**
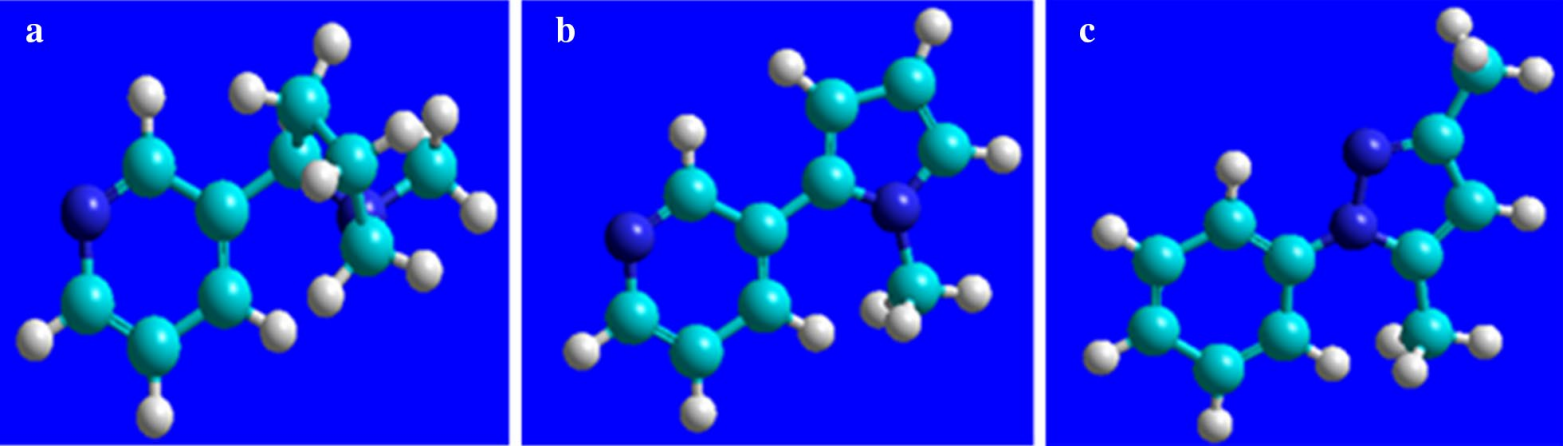
Models of the major alkaloids investigated in this work. **a** is nicotine, **b** is β-nicotyrine, while **c** is 3,5-dimethyl-1-phenylpyrazole

Numerous ailments as a consequence of tobacco use continue to decimate the human population. Inevitably, cigarette smoking has claimed so many lives despite intense research in this body of work. For instance, more than 5 million deaths per year have been attributed to tobacco use worldwide, and statistics predict that by 2030 in excess of 8 million deaths per year will be associated with tobacco consumption [1]. This study reports for the first time the thermochemistry of some of the tobacco alkaloids never accorded serious attention before in literature; β-nicotyrine and 3,5-dimethyl-1-phenylpyrazole. Additionally, the most studied alkaloid (nicotine) which is widely believed to be the cause of addiction in cigarette smoking has been thoroughly investigated. Pyrrole and pyridine are also investigated in this work. Whereas nicotine is the most abundant alkaloid, accounting for approximately 95 % of alkaloid content, the other alkaloids (β-nicotyrine, 3,5-dimethyl-1-phenylpyrazole, pyridine, and 3-methylpyridine) have been shown to exhibit biological activity resulting to serious cellular damage, heart disease, and respiratory illnesses [1]. In the United States alone, one out of every five deaths is initiated by cigarette smoking and this remains the foremost cause of preventable death with approximately 443,000 deaths per year [1, 2]. The major alkaloids investigated in this work are presented in Fig. 1, and modeled using HyperChem [3].

The study gives a detailed mechanistic description of the formation of common alkaloid-based radicals in tobacco smoke which are usually considered injurious to the biological health of smokers. Special interest is given to the thermochemistry of the formation of free radicals and other by-products of tobacco (pyridine, 3-methylpyrdine, toluene, and benzene). The toxicology of molecular reaction products (alkaloids) and their corresponding free radicals is discussed based on our results and literature data. We believe this study is fundamental towards unraveling some of the mechanistic pathways of alkaloids in mainstream cigarette smoking. Consequently, various competing pathways for major alkaloid transformation to various intermediates and molecular products have been investigated. Moreover, the bond dissociation energies for aliphatic linkages of major alkaloids and radical formation have thoroughly been presented. The understanding of the mechanistic destruction of the major alkaloid will widen our knowledge on their energetics, the formation of free radicals, intermediates, and their environmental fate.

## Experimental procedure

### Materials

The heater (muffle furnace) was purchased from Thermo Scientific Inc., USA while the quartz reactor was locally fabricated in our laboratory by a glass-blower. Commercial cigarettes SM1 and ES1 (for confidentiality) were purchased from retail outlets and used without further treatment. Methanol (purity ≥99 %) used to dissolve cigarette pyrolysate was purchased from Sigma Aldrich Inc. (USA).

### Sample preparation

50 mg of tobacco was packed in a quartz reactor of dimensions: i.d. 1 cm × 2 cm (volume $\approx$1.6 cm^3^). The tobacco sample in the quartz reactor was placed in an electrical heater furnace whose maximum heating temperature is 1000 °C with heating rate of ~20 °C/s. The tobacco sample was heated in flowing nitrogen (pyrolysis gas) to maintain a residence time of 2.0 s and the smoke effluent was allowed to pass through a transfer column and collected in 10 mL methanol in a conical flask for a total pyrolysis time of 2 min and sampled into a 2 mL crimp top amber vials for GC–MS analysis. This combustion experiment was conducted under conventional pyrolysis described elsewhere [4] and the evolution of pyridine, 3-methylpyridine, toluene, and benzene were monitored between 200 and 700 °C. All the data reported in this study are averaged replicates of two data points.

### GC–MS identification of tobacco alkaloids

GC–MS analysis was carried out using an Agilent Technologies 7890A GC system coupled with an Agilent Technologies 5975C inert XL electron ionization/chemical ionization (EI/CI) with a triple axis mass selective detector, using HP-5MS 5 % phenyl methyl siloxane column (30 m × 250 µm × 0.25 µm). The temperature of the injector port was set at set at 200 °C to enable the conversion of organic components to the gas-phase prior to MS analysis. The carrier gas was ultra-high pure (UHP) helium (99.999 %). The flow rate of the carrier gas (He) was set at 3.3 mL/min at 1 atmosphere pressure. Temperature programming was applied at a heating rate of 15 °C for 10 min, holding for 1 min at 200 °C, followed by a heating rate of 25 °C for 4 min, and holding for 10 min at 300 °C. Electron Impact ionization energy of 70 eV was used. The data was run through the NIST library database as an additional tool to confirm the identity of compounds [4]. To ensure that the right compound was detected, standards were run through the GC–MS system and the peak shapes and retention times compared with the compounds of interest.

### Computational methodology

The use of molecular modeling plays a critical role in the environmental regulatory processes. This is because complex relationship between environmental emissions, the quality of the environment, and human and toxicological impacts can be clearly described by computational procedures [5]. Density functional theory (DFT) optimizations at B3LYP/6-31G quantum level have been performed on all the molecular compounds as well as their respective free radicals. All thermochemical calculations have been carried out using Gaussian '09 [6–8]. Nevertheless, when using DFT, the choice of basis set is considered to be insignificant because the convergence of DFT to the basis-set limit with increasing size of basis set is relatively quick, thus small basis sets are preferred [9]. More often, diffuse functions on basis sets are not used for DFT calculations, since they lead to linear dependencies and a poor convergence of the self-consistent-field (SCF) Kohn–Sham equations for larger molecules [9]. Despite continuing improvements in formulating new DFT functionals with advanced predictive capabilities, the B3LYP functional retains its comparative accuracy in general applications to organic systems [5]. Chemissian *ver.4.38* computational software was used to model molecular orbital energy level diagrams, electron density maps as well as determine the band gap energies of frontier orbitals (HOMO–LUMO) [9, 10] for the selected tobacco alkaloids.

## Results and discussion

### Mechanistic pathways for radical formation and other possible molecular products

**Scheme 1:**
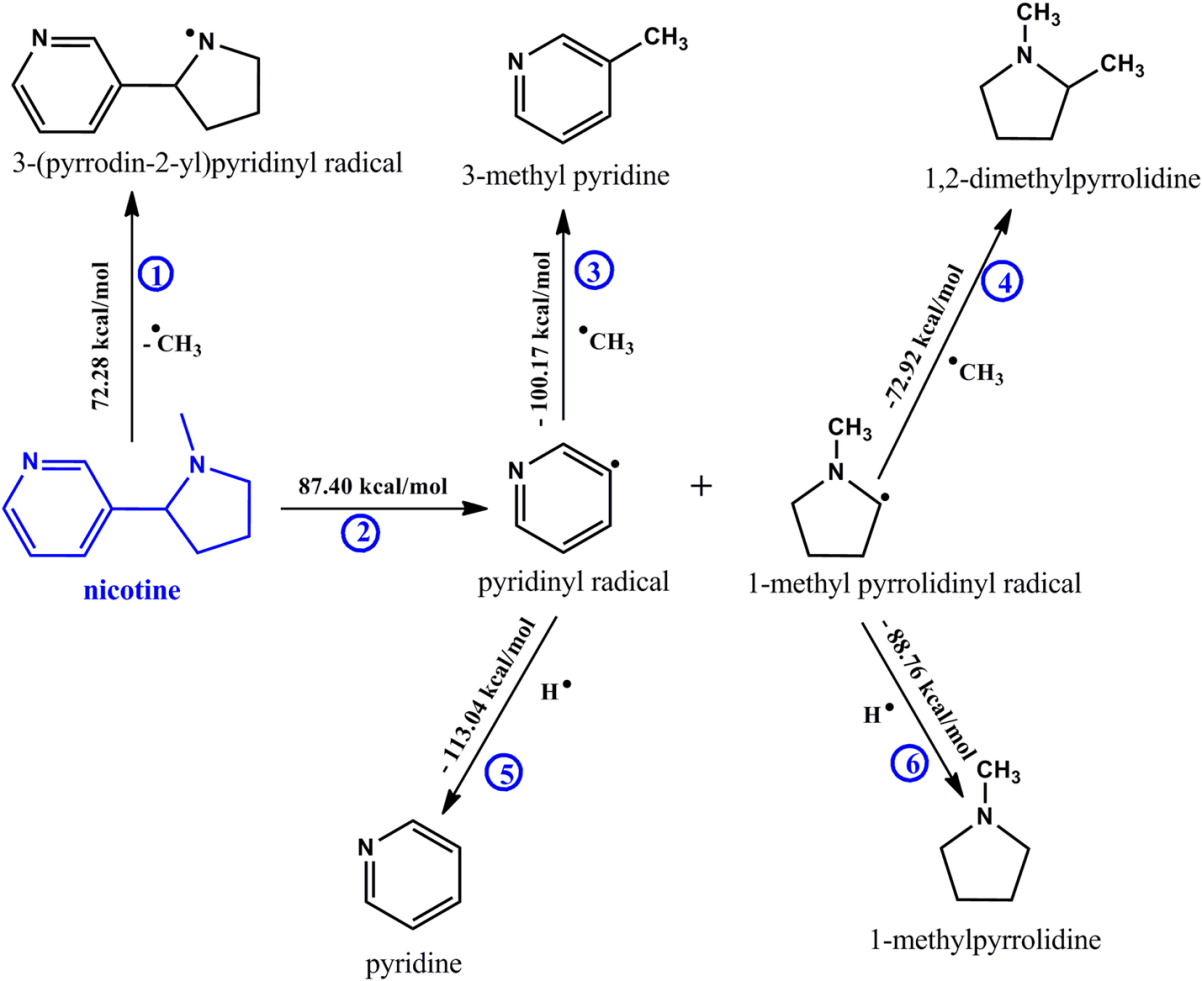
Proposed mechanistic pathways for the thermal degradation of nicotine

**Scheme 2:**
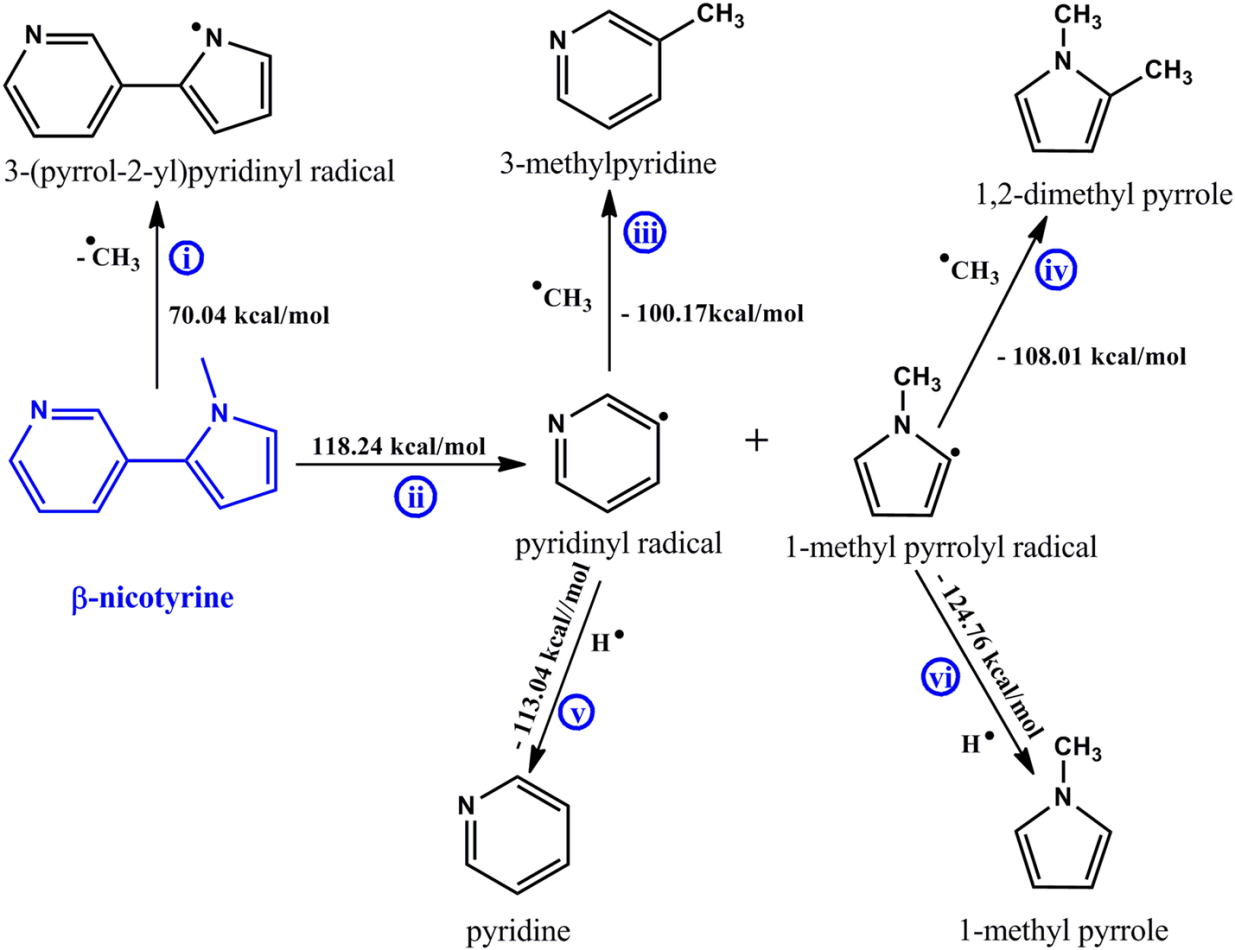
Proposed mechanistic pathways for the thermal degradation of β-nicotyrine

**Scheme 3:**
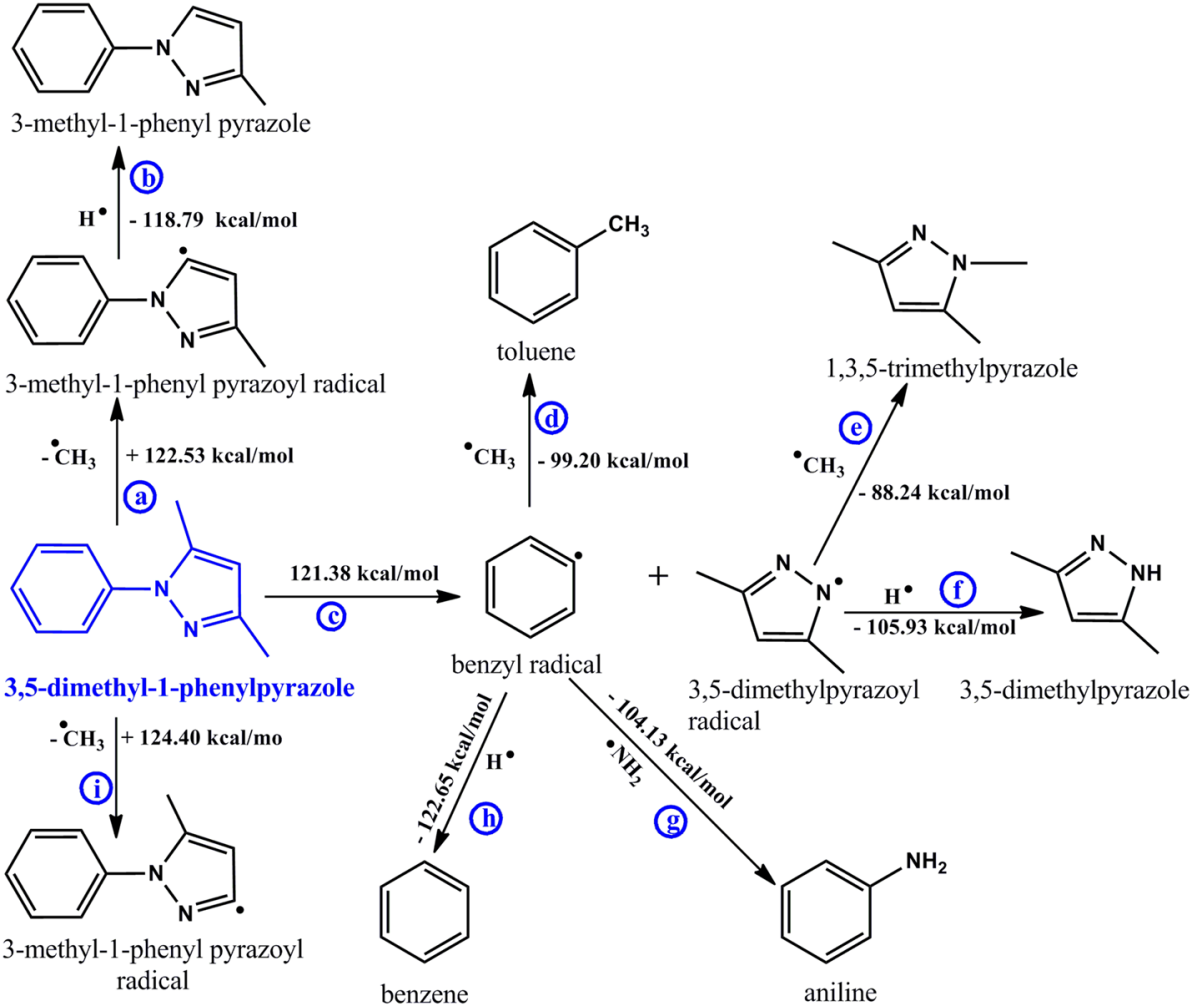
Proposed mechanistic pathways for the thermal degradation of 3,5-dimethyl-1-phenyl pyrazole

A meticulous description for the transformation of major tobacco alkaloids to their corresponding free radicals, intermediate by-products, and other possible alkaloids in tobacco has been explored using the density functional theory with the B3LYP hybrid correlation function in conjunction with the 6-31G basis set [5, 9]. Competing mechanistic pathways have been investigated and interesting data presented. The molecules in blue (herein referred to as the reactants, Schemes 1, 2 and 3) are the proposed starting alkaloids for the formation of various species indicated in the schematic reaction channels presented in this study. All computational calculations were conducted at a modest reaction temperature of 298.15 K at 1 atmosphere.

Clearly in all the schemes presented in this work (vide infra), there are several competing reaction pathways accompanied by different enthalpic barriers. For example in Scheme 1, Rxns 1 and 2 are the primary competing pathways for the thermal degradation of nicotine. Rxns 3 and 4, 5 and 6 are the other competing pathways for the transformation of intermediate radicals (pyridinyl and 1-methylpyrrolidinyl respectively).

### The proposed mechanistic channel for the thermal degradation of nicotine

**Fig. 2:**
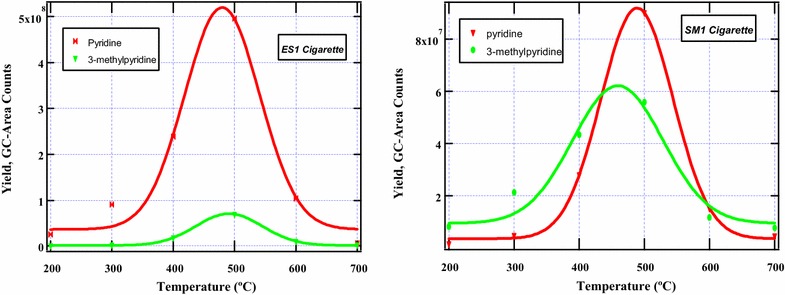
Product distribution of pyridine and 3-methypyridine in mainstream cigarette smoke determined from the burning of commercial cigarettes; ES1 (*left*) and SM1 (*right*)

This study investigates the possible mechanistic pathways involved during the thermal degradation of nicotine in tobacco burning to various intermediates and by-products. Clearly, the loss of a methyl group (Rxn 1) is accompanied by a less endothermic energy (72.28 kcal/mol) as compared to Rxn 2 which proceeds with a modest endothermic energy of 87.40 kcal/mol). Whereas Rxn 1 is expected to take place with minimum absorption of energy, it leads to the formation of few major intermediates; 3-(pyrrodin-2-yl)pyridinyl radical and possibly 3-(pyrrolidin-2-yl) pyridine. Thus these routes will not be examined further considering the fact that 3-(pyrrolidin-2-yl) pyridine was not detected in mainstream cigarette smoke in the two cigarettes investigated. This leaves us to consider Rxn 2 which results into many intermediates some of which were detected experimentally in our studies. The scission of the phenyl-cyclopenta C–C bond in nicotine which yields pyridinyl and 1-methylpyrrolidinyl radical proves a very important pathway. Interestingly, Rxns 3 and 5 are competing reaction channels for the formation of neutral species (3-methylpyridine and pyridine respectively). Since the hydride radical is more reactive than the methyl radical according to previous studies [11], then the formation of pyridine is expected to be formed in larger amounts than 3-mthylpyridine. This observation is consistent with the results obtained in this work. Accordingly, the product distribution of pyridine and 3-methylpyridine as a function of smoking temperatures has been presented in Fig. 2 to validate the computational results determined in this study.

Pyridine, nevertheless was found to be high in ES1 cigarette and low in SM1 cigarette while the concentrations of 3-methylpyridine were comparable in the two cigarettes (cf. Fig. 2). On the other hand, Rxns 4 and 6 are the other two parallel pathways. Although the formation of 1,2-dimethylpyrrolidine and 1-methylpyrrolidine were detected in low amounts in mainstream cigarette smoke for the two cigarettes under study, it is clear that Rxn 6 proceeds with high exothermicity (−88.76 kcal/mol) and possibly more favourable than Rnx 4 which proceeds with an enthalpic change of −72.92 kcal/mol. The low exothermic value in Rxn 4 may be attributed to the low reactivity of the methyl radical ($^{ \bullet } CH_{3}$) in comparison to the reactivity of the hydride radical.

### Mechanistic description for the thermal degradation of β-nicotyrine

Although it may appear the molecular structure of β-nicotyrine and that of nicotine are similar, their chemistries are significantly different because of the influence of the C–C double bonds in the cyclopenta ring which are absent in nicotine. This results in high bond dissociation energy for the phenyl/cyclopenta C–C bond presented by Rxn *ii* (118.24 kcal/mol) compared to Rxn 2 in Scheme 1, vide supra and Rnx *c*, Scheme 3, vide infra. Another interesting reaction is Rxn *i* which proceeds with an endothermicity of 70.04 kcal/mol compared to Rxn 1 which takes place with and absorption of 72.28 kcal/mol (Scheme 1).

The parallel reaction *iii* and *v* are very similar to reactions 3 and 5 in Scheme 1, and will not be the subject of further discussion. However, Scheme 1 and 2 are responsible for the observed levels of pyridine and 3-methylpyridine in cigarette smoke presented in Fig. 2. The formation of methylated pyrroles from 1-methylpyrrolyl radical is presented by the parallel reactions *iv* and *vi*. As previously discussed, the H radical is very reactive compared to the CH_3_ radical and therefore, 1-methylpyrrole will be expected to be formed in significant amounts in tobacco smoke. This result agrees well with our experimental results in which 1-methylpyrrole though a minor product was detected in significant amounts as compared to 1,2-dimethylpyrrole.

### The mechanistic pathway for decomposition of 3, 5-dimethyl-1-phenylpyrazole

**Fig. 3:**
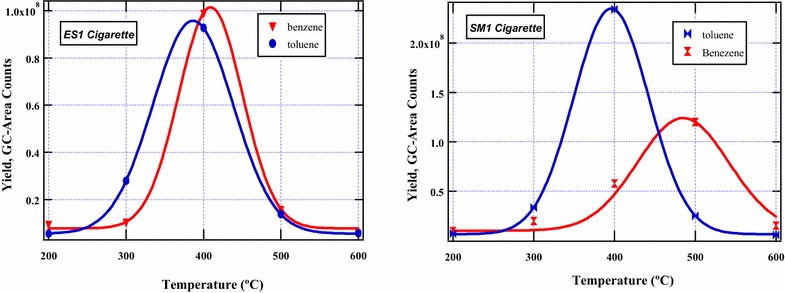
Product distribution of toluene and benzene in mainstream cigarette smoke determined from the burning of commercial cigarettes; ES1 (*left*) and SM1 (*right*)

The chemistry of 3,5-dimethyl-1-phenyl pyrazole is quite remarkable because its decomposition during cigarette smoking is predicted to yield several intermediate as well as stable by-products. The most important reaction products which were detected in significant amounts experimentally were toluene, benzene, and aniline which have successfully been predicted computationally in this scheme. Despite the fact that toluene and benzene are not the focus of this study, their products yields are presented in Fig. 3 to qualify the theoretical explanations presented in Scheme 3. Whereas the molecular structures of nicotine and β-nicotyrine contain a nitrogen atom in the phenyl ring, 3,5-dimethyl-1-phenylpyrazole does not contain a nitrogen atom. This explains why 3,5-dimethyl-1-phenylpyrazole can easily form aromatic hydrocarbons (benzene and toluene) while nicotine and β-nicotyrine do not. Nevertheless, the bond dissociation energy for the phenyl C–N bond in nicotine (72.28 kcal/mol, Rnx 1) is much lower than in 3,5-dimethyl-1-phenylpyrazole (122.53 kcal/mol, Rxn *a*) according to Schemes 1 and 3 respectively. The ratio between the two energies is ~1.7 indicating that the C–C double bonds and the phenyl nitrogen bond have a significant influence on the C–N bond in 3,5-dimethyl-1-phenylpyrazole. These groups are electron donating and therefore stabilize the methyl attached to the cyclopenta group in 3,5-dimethyl-1-phenylpyrazole. This makes it difficult for the $^{ \bullet } CH_{3}$ to leave during the pyrolysis. The scission of the methyl group in Rxn *i* (Scheme 3) occurs with higher endothermicity (124.40 kcal/mol). This again is attributed to the electron rich C–C double bond and the C–N bonds adjacent to the methyl which are electron donating and thus the methyl is strongly stabilized. The transformation of benzyl radical to molecular products (toluene, benzene, aniline) proceeds via three parallel pathways: Rxn *d* (−99.20 kcal/mol), Rxn *h* (−112.65 kcal/mol), and Rxn *g* (−104.13 kcal/mol).

Clearly, the formation of benzene from benzyl radical (Rxn *h*) is the most preferred pathway because the hydride radical is more reactive than both the amine radical (Rxn *g*) as well as the methyl radical (Rxn *d*). Nonetheless, the amount of toluene formed from the combustion of the SM1 cigarette was found to be more than that of benzene implying that there might be other pyrosynthetic pathways in tobacco that result in the formation of toluene. Such possible pathways will not be the subject of this investigation.

Remarkably, the amount of toluene and benzene evolved for the ES1 cigarette were found to be similar according to Fig. 3. To qualify the mechanistic description for the formation of benzene and toluene, product evolution curves of these compounds are presented in Fig. 6, vide infra. Similar explanations can be inferred for the competing reactions *e* and *f*. Although methylated pyrazoles were detected in low amounts in our experiments, it is evident that the addition of H radical to the intermediate 3,5-dimethylpyrazoyl radical is the most preferred mechanistic channel because of the high exothermicity of −105.93 kcal/mol.

Whereas toluene and benzene reach a maximum at about the same temperature (~400 °C) for ES1 cigarette, the evolution characteristics of toluene and benzene for SM1 cigarette vary markedly. For instance, toluene peaks at 400 °C while benzene peaks at about 500 °C. Nevertheless, since toluene and benzene are not the primary focus of this work, their molecular behaviour as well as their toxicities will not be discussed further.

### Molecular geometries of major tobacco alkaloids

**Fig. 4:**
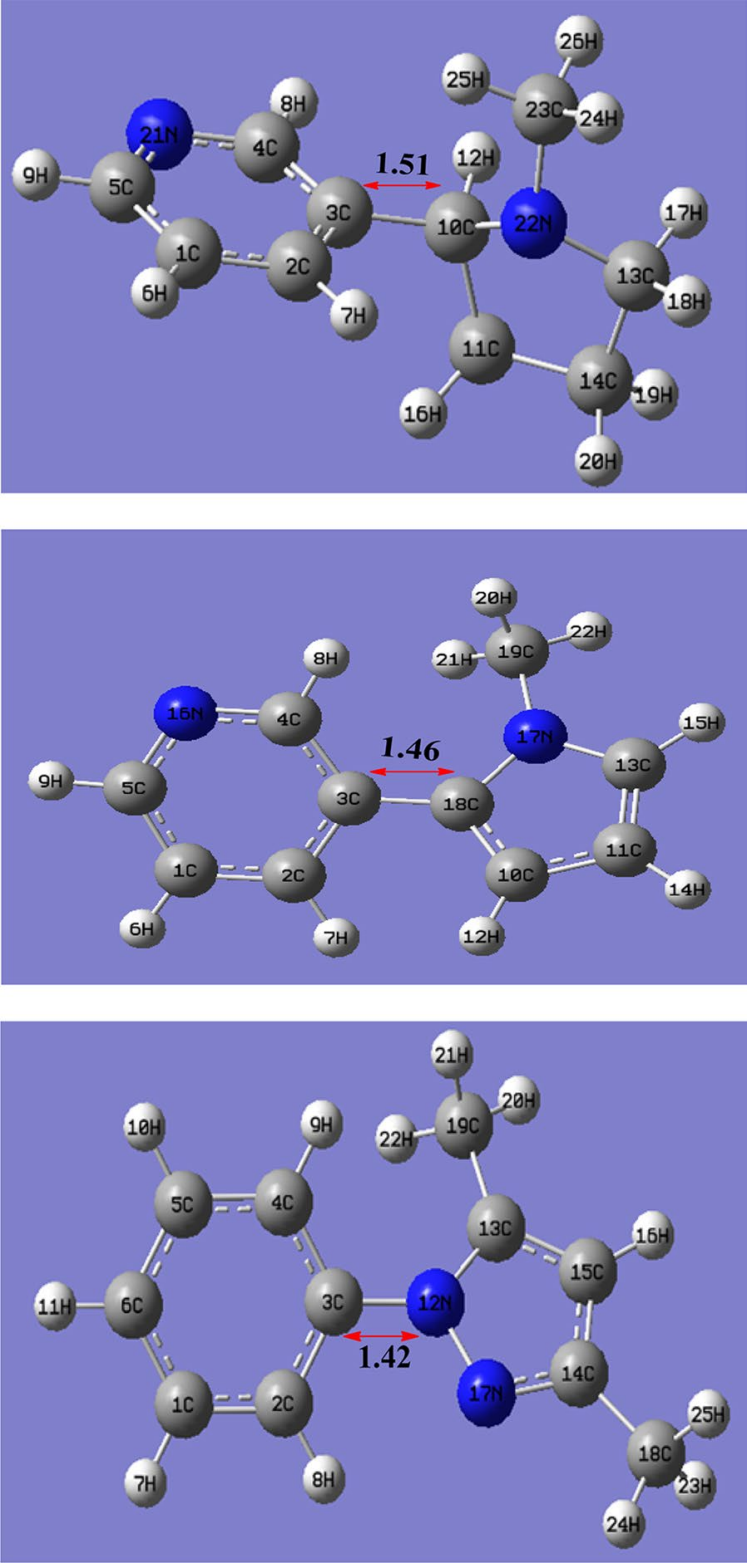
Comparison of bond-lengths of nicotine, β-nicotyrine and 3,5-dimethyl-1-phenylpyrazole (from *top* to *bottom* respectively). Bond lengths are given in Ȧ

Geometrical parameters such as bond lengths and bond angles have a great influence on the strength of the bonds of molecular structures [12]. Moreover, computational chemistry provides insight into the molecular properties of a compound that would not be easy to determine experimentally. Accordingly, the comparison of the optimized structures for the phenyl C–C bond lengths for nicotine and β-nicotyrine, and the phenyl C–N bond length for 3,5-dimethyl-1-phenylpyrazole has been presented in Fig. 4. Conventionally, from thermodynamic point of view, the bond-dissociation energy should increase with decrease in the bond length. This observation is remarkable and is consistent with our results.

The molecular geometries starting from the top of the page to the bottom are respectively nicotine, β-nicotyrine and 3,5-dimethyl-1-phenylpyrazole. The bond strength increases from nicotine to β-nicotyrine to 3,5-dimethyl-1-phenylpyrazole as presented in Fig. 4. This implies that, the shorter the bond length, the higher the bond dissociation energy. This is consistent with the thermochemical results presented in Schemes 1, 2 and 3. Accordingly, the bond dissociation energies increase in a similar fashion; the scission of 3C, 10C (1.51 Ȧ) bond in nicotine proceeds with an energy of 87.40 kcal/mol, while the bond C3, C18 (1.46 Å) in β-nicotyrine takes place with a bond dissociation energy of 118.24 kcal/mol. The scission of 3C, 12 N (1.42 Å) in 3,5-dimethyl-1-phenylpyrazole occurs with a bond dissociation energy of 121.38 kcal/mol. Evidently, the phenyl bond dissociation in β-nicotyrine and 3,5-dimethyl-1-phenylpyrazole are effectively stabilized by the π–π interactions than in nicotine molecule.

### Molecular orbitals and electron density maps of nicotine

The HOMO and the LUMO are conventional acronyms for the highest occupied and lowest unoccupied molecular orbitals respectively. These orbitals are the pair that lie nearest in energy of any pair of orbitals in any two molecules, which permits them to interact more strongly [8]. The HOMO–LUMO band-gap energies for the alkaloids under study are presented in Table 1. The reactivity index (band gap) of the compounds with small difference implies high reactivity and a large difference implies low reactivity in reactions, therefore as the energy gap between the HOMO and LUMO becomes smaller the rate of reaction is favoured. β-nicotyrine has the smallest HOMO–LUMO energy gap (4.811 eV) and therefore more reactive compared to nicotine (5.492 eV) and 3,5-dimethyl-1-phenylpyrazole (6.057 eV). Though 3,5-dimethyl-1-phenylpyrazole may have a large HOMO–LUMO energy gap it is considered a good electron donor [12].

**Table 1:** Band-gap energies for the alkaloids investigated in this work

Compound	HOMO (eV)	LUMO (eV)	ΔH = E_LUMO_−E_HOMO_ (eV)
Nicotine	−5.974	−0.482	5.492
β-nicotyrine	−5.837	−1.026	4.811
3,5-dimethyl-1-phenylpyrazole	−6.213	−0.156	6.057

**Fig. 5:**
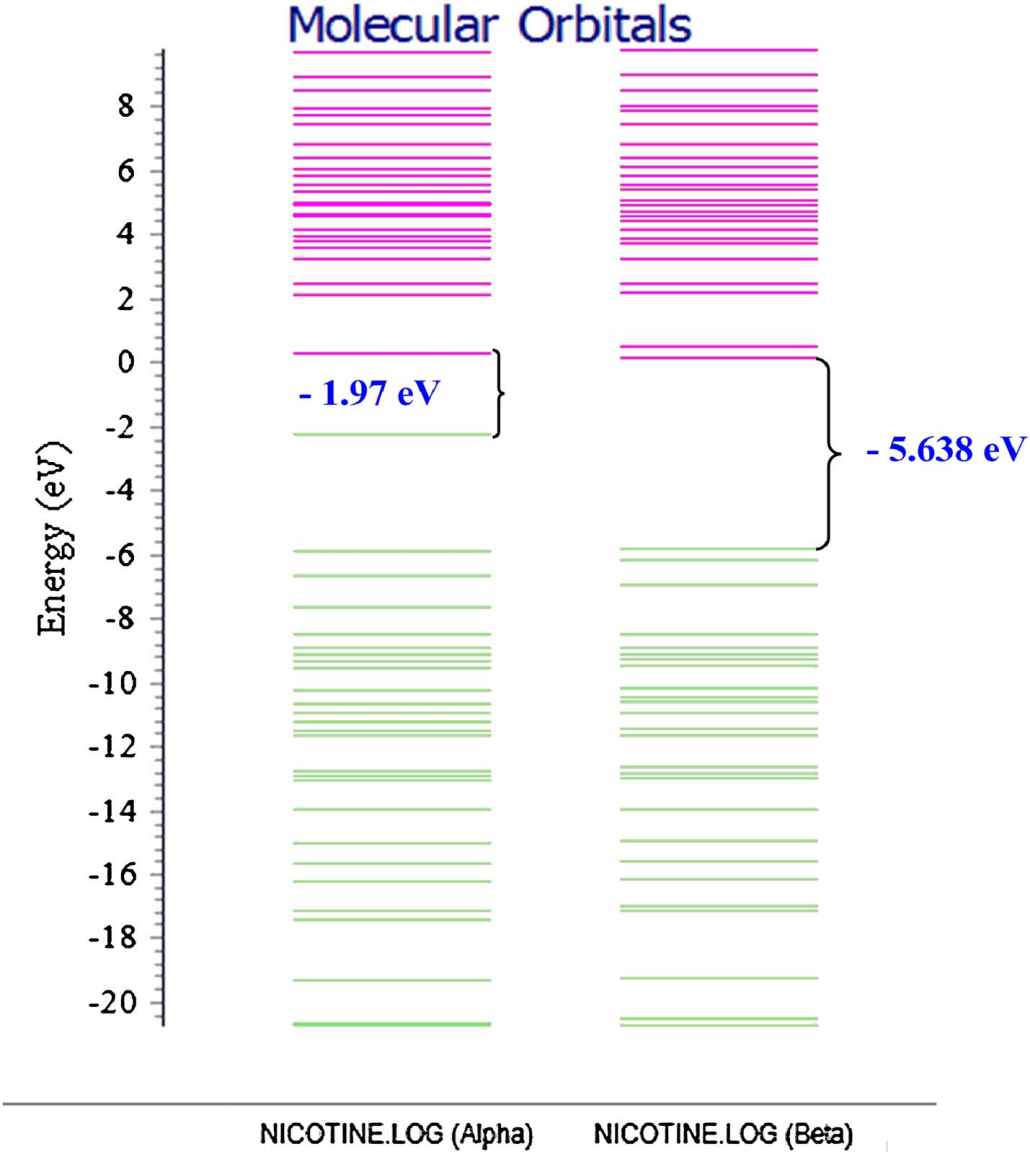
The HOMO–LUMO band gap for nicotine determined using Chemissian (α and β electron orientation)

**Fig. 6:**
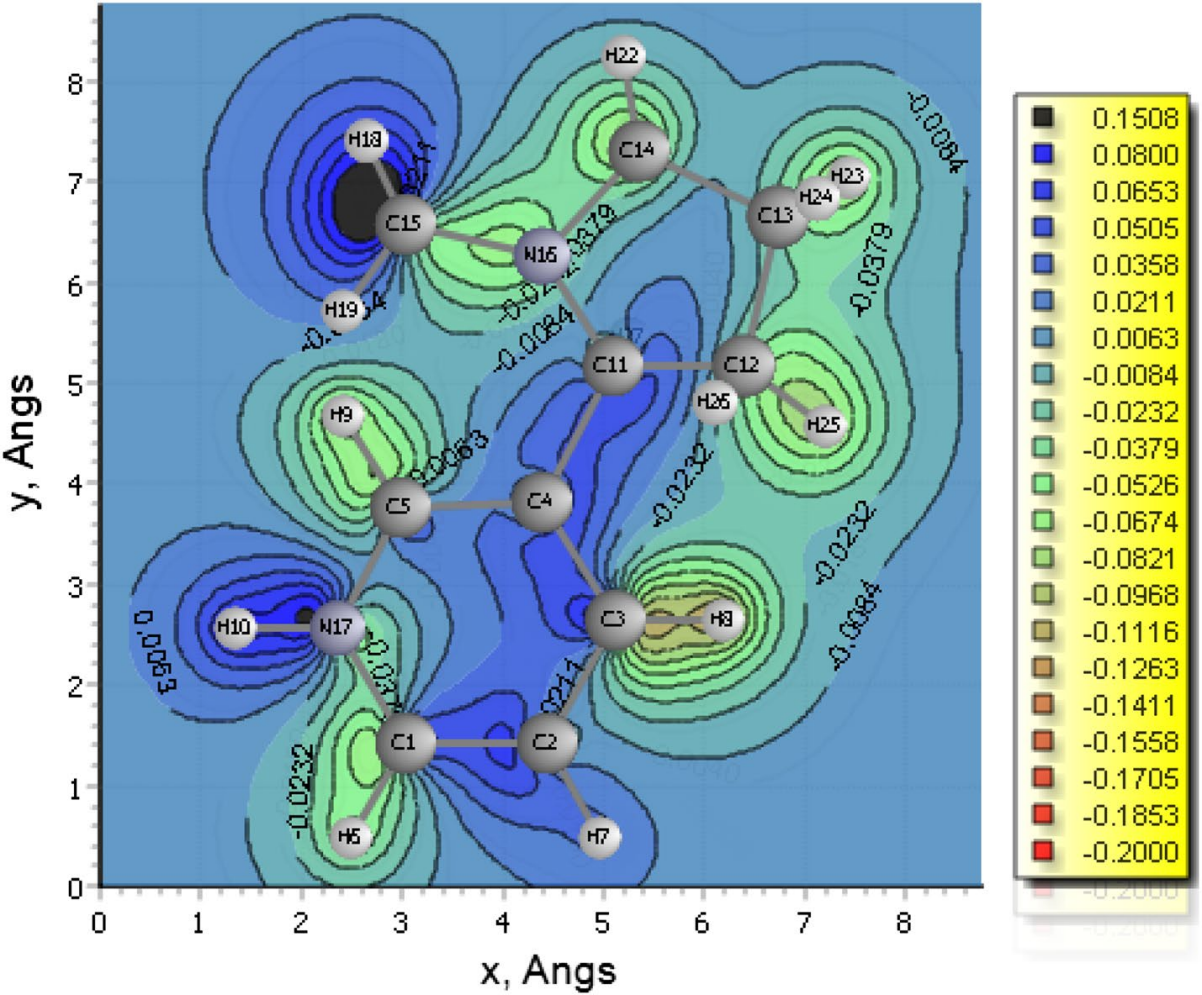
2-D electron density map for nicotine

**Fig. 7:**
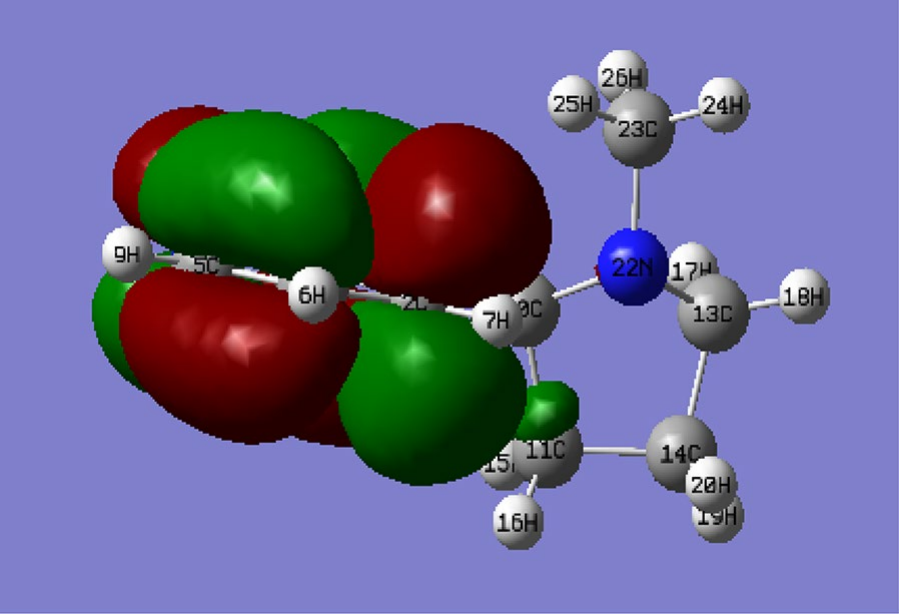
3-D molecular orbital diagram showing electronic density for nicotine at an isovalue of 0.02

**Fig. 8:**
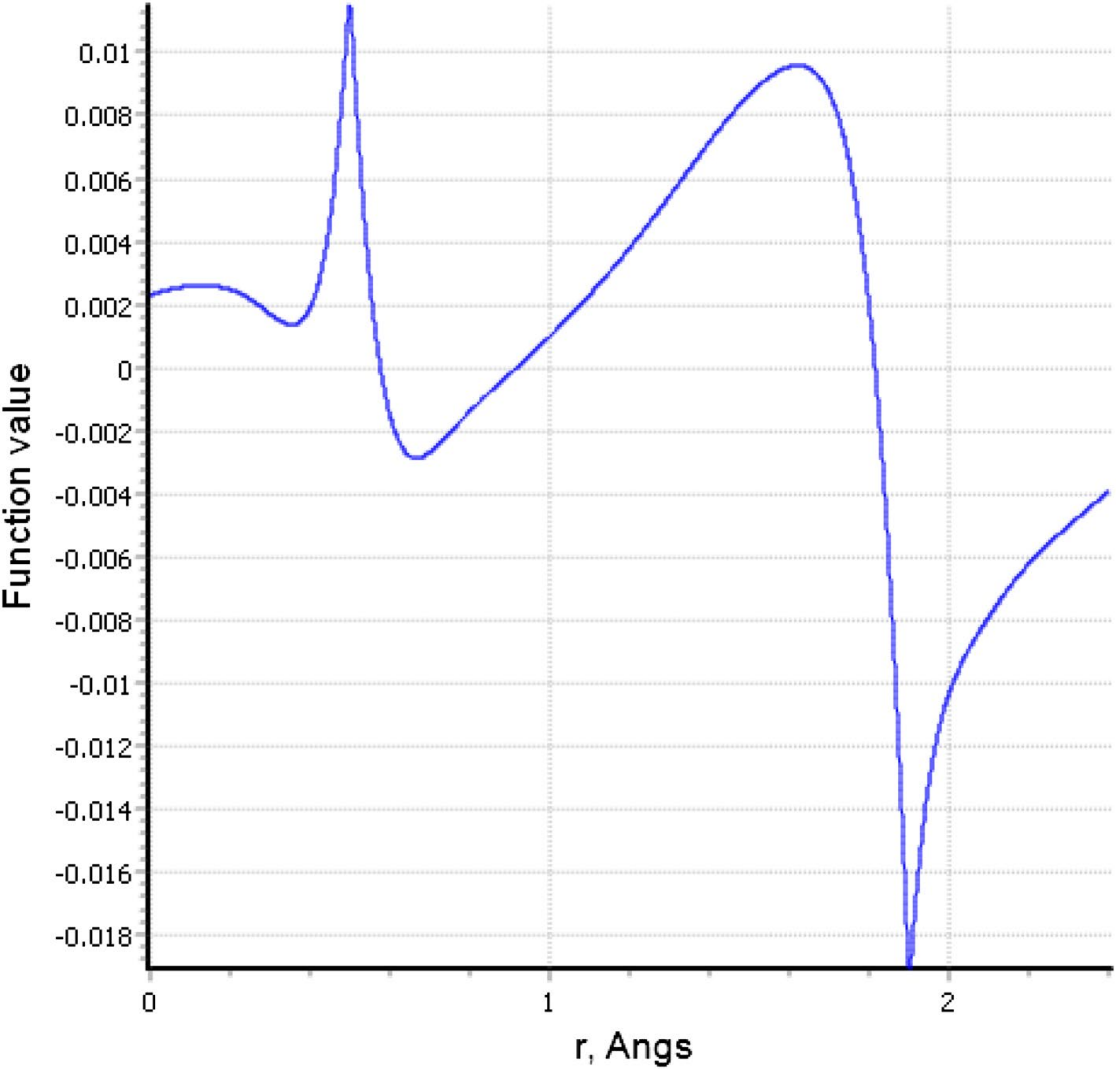
*1-D line* showing the probability of finding electrons at a distance r from nicotine nuclei

In this investigation, the HOMO–LUMO band-gap of nicotine and β-nicotyrine are significantly low and may be reactive especially towards biological structures. This may explain the fact that nicotine is immediately adsorbed into the blood stream and reaches the brain in 10–20 s seconds after a cigarette puff as reported in literature [13]. Generally, the band-gap between the HOMO and the LUMO is directly related to the electronic stability of the chemical species [12]. This suggests that 3,5-dimethyl-1-phenylpyrazole having a lower HOMO energy value of −6.213 eV is much more stable making it a good nucleophile compared to nicotine and β-nicotyrine which are energetically higher in the HOMO; −5.974 and −5.837 eV respectively. The application of Chemissian software facilitated the construction of electron density contour maps and molecular orbitals [10, 14] for nicotine (Figs. 5, 6, and 8, vide infra). Figure 7 has been modeled using Gaussian '09 computational code.

The electron density contours maps for 2D-, 3D-, and 1-dimensions for nicotine are presented in Figs. 5, 6, 7 and 8 respectively. Electron density maps are very important in understanding electrophilic and nucleophilic sites. Conventionally, The negative potential sites (red colour) represents regions of electrophilic reactivity and interactions through π–π bonding within aromatic systems and positive potential sites (green colour) represents regions of nucleophilic reactivity [12]. Similar electron density maps were done for β-nicotyrine and dimethyl-1-phenylpyrazole as presented in Additional file 1. These figures are critical in determining regions of high electron density within a molecule. Electron distribution gives insight on the behaviour of a particular toxicant and probably the binding site during reactions with biological molecules such as DNA, microsomes, and lipids.

### Possible health impacts of molecular alkaloids and their free radicals

Molecular products of tobacco including alkaloids may be metabolized primarily in the biological system to a series of ring-opened by-products which may cause severe alveoli and other cellular injuries [15, 16]. This makes alkaloid-based free radicals such as those presented in Schemes 1, 2 and 3; vide supra, potential clinical candidates for a variety of illnesses affecting cigarette smokers. The radicals generated during cigarette smoking are generally hazardous as they have the possibility of reacting with biological tissues such as DNA, lipids and lung microphages to initiate tumors, cancer and oxidative stress [16]. In this study, the radicals including pyridinyl, benzyl, 1-methyl pyrrolyl, and 1-methylpyrrolidinyl radicals are good candidates for cell damage and oxidative stress during cigarette smoking.

## Conclusion

This study has presented a thorough mechanistic description on the molecular characteristics of major alkaloids (nicotine, β-nicotyrine, and 3,5-dimethyl-1-phenylpyrazole) never articulated before in literature. The environmental fate of various intermediates from the major tobacco alkaloids have been discussed in detail in this work and this forms and important basis for understanding tobacco pollutants. Moreover, the consistency between experimental formation of pyridine, 3-methylpyridine, toluene, and benzene, and computational predictions is remarkable. It was also established that the strength of the C–C and C–N bonds in phenyl-cyclopenta linkages in the alkaloids investigated in this work were dependent on the π–π interactions which stabilize the bonds. Therefore because of the small bond dissociation energy required to break the phenyl C–C linkage in nicotine (87.40 kcal/mol) compared to 118. 24 kcal/mol required to break the C–C phenyl bond in β-nicotyrine, it is apparent that most of the yields of pyridine and 3-methylpyridine observed from our experiments are proposed to originate from the thermal degradation of nicotine.

## Additional file

10.1186/s13065-016-0189-5 Additional information.
