# Supplementary material for: Molecular modeling of major tobacco alkaloids in mainstream cigarette smoke
Source: Chem Cent J. 2016 Jul 15;10:43. doi: 10.1186/s13065-016-0189-5 (PMC4947291; doi:10.1186/s13065-016-0189-5)
Supplement: Supplementary file 1 — 10.1186/s13065-016-0189-5 Additional information. [file 13065_2016_189_MOESM1_ESM.docx]

**Molecular Modeling of Major Tobacco Alkaloids in Mainstream Cigarette Smoke**

Caren Kurgat, Joshua Kibet* and Peter Cheplogoi

Department of Chemistry, Egerton University P.O Box 536 -20115, Egerton, Kenya

*Corresponding author: [jkibet@egerton.ac.ke](mailto:jkibet@egerton.ac.ke) Tel: +254 720 352 437

**Supporting information**

**S1: Frequency and thermochemistry output file for optimization of pyridinyl radical**

Full mass-weighted force constant matrix:

Low frequencies --- -19.1100 -13.9534 -0.0009 -0.0007 -0.0003 2.0451

Low frequencies --- 399.0217 438.0293 595.5201

Diagonal vibrational polarizability:

1.1043663 1.5610057 3.6797222

Harmonic frequencies (cm**-1), IR intensities (KM/Mole), Raman scattering

activities (A**4/AMU), depolarization ratios for plane and unpolarized

incident light, reduced masses (AMU), force constants (mDyne/A),

and normal coordinates:

1 2 3

A A A

Frequencies -- 399.0212 438.0291 595.5201

Red. masses -- 2.7803 4.2158 7.8729

Frc consts -- 0.2608 0.4766 1.6450

IR Inten -- 2.8698 0.0063 18.7184

Atom AN X Y Z X Y Z X Y Z

1 6 0.00 0.00 0.24 0.00 0.00 -0.04 0.17 0.23 0.00

2 6 0.00 0.00 -0.12 0.00 0.00 0.24 -0.30 0.27 0.00

3 6 0.00 0.00 -0.13 0.00 0.00 -0.33 -0.18 -0.13 0.00

4 6 0.00 0.00 0.21 0.00 0.00 0.00 -0.18 -0.18 0.00

5 6 0.00 0.00 -0.12 0.00 0.00 -0.21 0.15 0.10 0.00

6 1 0.00 0.00 0.56 0.00 0.00 0.03 0.28 -0.01 0.00

7 1 0.00 0.00 -0.27 0.00 0.00 0.71 -0.34 0.22 0.00

8 1 0.00 0.00 0.59 0.00 0.00 0.15 -0.30 0.14 0.00

9 1 0.00 0.00 -0.33 0.00 0.00 -0.45 -0.22 0.13 0.00

10 7 0.00 0.00 -0.11 0.00 0.00 0.26 0.34 -0.28 0.00

4 5 6

A A A

Frequencies -- 675.1487 704.6629 803.3418

Red. masses -- 7.2797 2.2120 1.5116

Frc consts -- 1.9551 0.6471 0.5748

IR Inten -- 2.7961 34.3180 31.0437

Atom AN X Y Z X Y Z X Y Z

1 6 0.33 0.01 0.00 0.00 0.00 -0.15 0.00 0.00 0.04

2 6 0.14 0.09 0.00 0.00 0.00 0.07 0.00 0.00 0.12

3 6 -0.02 0.43 0.00 0.00 0.00 -0.12 0.00 0.00 -0.10

4 6 -0.31 -0.04 0.00 0.00 0.00 0.12 0.00 0.00 0.08

5 6 0.03 -0.34 0.00 0.00 0.00 0.13 0.00 0.00 0.09

6 1 0.22 0.25 0.00 0.00 0.00 0.08 0.00 0.00 -0.75

7 1 -0.14 -0.29 0.00 0.00 0.00 0.51 0.00 0.00 -0.51

8 1 -0.25 -0.20 0.00 0.00 0.00 0.49 0.00 0.00 -0.15

9 1 -0.03 -0.34 0.00 0.00 0.00 0.62 0.00 0.00 -0.34

10 7 -0.13 -0.09 0.00 0.00 0.00 -0.17 0.00 0.00 -0.07

7 8 9

A A A

Frequencies -- 937.2052 962.4442 992.1842

Red. masses -- 1.3440 1.3482 8.6355

Frc consts -- 0.6955 0.7358 5.0087

IR Inten -- 0.7314 0.2507 5.3297

Atom AN X Y Z X Y Z X Y Z

1 6 0.00 0.00 0.03 0.00 0.00 0.03 0.42 0.14 0.00

2 6 0.00 0.00 0.06 0.00 0.00 -0.10 -0.07 0.09 0.00

3 6 0.00 0.00 0.02 0.00 0.00 0.02 -0.04 -0.43 0.00

4 6 0.00 0.00 -0.15 0.00 0.00 -0.07 0.02 -0.11 0.00

5 6 0.00 0.00 -0.04 0.00 0.00 0.12 0.08 -0.02 0.00

6 1 0.00 0.00 -0.21 0.00 0.00 -0.29 0.53 -0.06 0.00

7 1 0.00 0.00 -0.38 0.00 0.00 0.58 -0.07 0.07 0.00

8 1 0.00 0.00 0.84 0.00 0.00 0.39 0.05 -0.20 0.00

9 1 0.00 0.00 0.27 0.00 0.00 -0.63 0.13 -0.02 0.00

10 7 0.00 0.00 0.03 0.00 0.00 -0.01 -0.39 0.30 0.00

10 11 12

A A A

Frequencies -- 1012.9335 1054.4541 1075.7611

Red. masses -- 1.4252 4.9885 2.0287

Frc consts -- 0.8616 3.2680 1.3832

IR Inten -- 0.3689 3.0776 6.8617

Atom AN X Y Z X Y Z X Y Z

1 6 0.00 0.00 -0.14 0.09 0.05 0.00 0.04 -0.06 0.00

2 6 0.00 0.00 0.08 0.25 -0.27 0.00 -0.14 0.00 0.00

3 6 0.00 0.00 0.00 -0.02 -0.02 0.00 0.03 0.16 0.00

4 6 0.00 0.00 -0.04 -0.25 -0.10 0.00 0.13 -0.08 0.00

5 6 0.00 0.00 0.11 -0.02 0.38 0.00 0.00 0.12 0.00

6 1 0.00 0.00 0.69 0.16 -0.03 0.00 0.27 -0.55 0.00

7 1 0.00 0.00 -0.41 0.13 -0.45 0.00 -0.37 -0.30 0.00

8 1 0.00 0.00 0.13 -0.30 -0.01 0.00 0.27 -0.46 0.00

9 1 0.00 0.00 -0.55 -0.34 0.42 0.00 -0.11 0.13 0.00

10 7 0.00 0.00 0.00 -0.01 -0.04 0.00 -0.06 -0.03 0.00

13 14 15

A A A

Frequencies -- 1121.9766 1219.5640 1276.2711

Red. masses -- 2.0586 1.2864 5.3813

Frc consts -- 1.5268 1.1273 5.1644

IR Inten -- 1.0375 2.2694 0.9345

Atom AN X Y Z X Y Z X Y Z

1 6 0.09 0.12 0.00 -0.04 0.02 0.00 -0.11 0.17 0.00

2 6 -0.04 -0.11 0.00 -0.02 0.00 0.00 -0.12 -0.13 0.00

3 6 -0.03 -0.03 0.00 0.00 0.01 0.00 0.18 -0.02 0.00

4 6 0.13 0.16 0.00 0.00 -0.12 0.00 -0.12 0.22 0.00

5 6 -0.07 -0.04 0.00 0.05 0.02 0.00 0.34 0.04 0.00

6 1 -0.06 0.46 0.00 -0.31 0.58 0.00 -0.04 0.01 0.00

7 1 -0.37 -0.56 0.00 -0.30 -0.38 0.00 0.16 0.25 0.00

8 1 0.12 0.19 0.00 0.05 -0.26 0.00 0.09 -0.39 0.00

9 1 -0.43 -0.03 0.00 0.48 0.00 0.00 0.61 0.01 0.00

10 7 0.00 -0.08 0.00 0.02 0.07 0.00 -0.21 -0.23 0.00

16 17 18

A A A

Frequencies -- 1361.6780 1453.0787 1480.5391

Red. masses -- 1.2795 1.8971 1.7449

Frc consts -- 1.3978 2.3600 2.2535

IR Inten -- 1.7391 19.1404 6.2261

Atom AN X Y Z X Y Z X Y Z

1 6 -0.04 0.00 0.00 -0.09 0.01 0.00 0.02 -0.06 0.00

2 6 0.01 0.07 0.00 -0.01 0.04 0.00 -0.11 -0.06 0.00

3 6 0.12 -0.02 0.00 0.14 -0.06 0.00 0.06 0.08 0.00

4 6 -0.02 -0.02 0.00 -0.12 0.03 0.00 0.05 -0.12 0.00

5 6 -0.02 0.00 0.00 0.09 -0.07 0.00 0.12 0.03 0.00

6 1 0.09 -0.28 0.00 -0.04 -0.14 0.00 -0.21 0.41 0.00

7 1 -0.34 -0.40 0.00 -0.26 -0.29 0.00 0.15 0.30 0.00

8 1 -0.28 0.68 0.00 0.02 -0.37 0.00 -0.20 0.56 0.00

9 1 0.28 -0.02 0.00 -0.78 -0.04 0.00 -0.51 0.06 0.00

10 7 -0.04 -0.02 0.00 0.06 0.11 0.00 -0.06 0.03 0.00

19 20 21

A A A

Frequencies -- 1545.9227 1612.2957 3200.3479

Red. masses -- 4.7784 6.0946 1.0860

Frc consts -- 6.7283 9.3344 6.5534

IR Inten -- 7.2771 7.1928 1.1564

Atom AN X Y Z X Y Z X Y Z

1 6 -0.09 0.38 0.00 -0.21 0.11 0.00 -0.05 -0.03 0.00

2 6 -0.07 -0.28 0.00 0.28 0.12 0.00 0.04 -0.02 0.00

3 6 0.01 0.10 0.00 -0.37 0.02 0.00 0.00 0.00 0.00

4 6 0.03 -0.18 0.00 0.17 -0.09 0.00 0.00 0.00 0.00

5 6 -0.05 -0.16 0.00 0.33 -0.04 0.00 0.00 0.04 0.00

6 1 0.37 -0.57 0.00 -0.02 -0.36 0.00 0.64 0.30 0.00

7 1 0.26 0.14 0.00 -0.10 -0.40 0.00 -0.41 0.30 0.00

8 1 -0.09 0.18 0.00 0.07 0.22 0.00 -0.02 -0.01 0.00

9 1 0.19 -0.19 0.00 -0.43 -0.03 0.00 -0.03 -0.47 0.00

10 7 0.09 0.15 0.00 -0.14 -0.06 0.00 0.00 0.00 0.00

22 23 24

A A A

Frequencies -- 3211.9845 3226.3963 3237.3069

Red. masses -- 1.0908 1.0971 1.0929

Frc consts -- 6.6301 6.7287 6.7483

IR Inten -- 15.7632 23.5847 5.5702

Atom AN X Y Z X Y Z X Y Z

1 6 0.00 -0.01 0.00 -0.06 -0.03 0.00 0.01 0.01 0.00

2 6 -0.05 0.04 0.00 -0.03 0.03 0.00 0.01 -0.01 0.00

3 6 0.00 0.00 0.00 0.00 0.00 0.00 0.00 0.00 0.00

4 6 0.00 0.00 0.00 -0.02 -0.01 0.00 -0.08 -0.03 0.00

5 6 0.00 0.06 0.00 0.00 -0.05 0.00 0.00 0.02 0.00

6 1 0.07 0.04 0.00 0.61 0.28 0.00 -0.15 -0.07 0.00

7 1 0.59 -0.44 0.00 0.34 -0.26 0.00 -0.09 0.07 0.00

8 1 -0.02 -0.01 0.00 0.25 0.09 0.00 0.90 0.33 0.00

9 1 -0.04 -0.67 0.00 0.02 0.53 0.00 -0.01 -0.18 0.00

10 7 0.00 0.00 0.00 0.00 0.00 0.00 0.00 0.00 0.00

-------------------

- Thermochemistry -

-------------------

Temperature 298.15 Kelvin. Pressure 1.00000 Atm.

Atom 1 has atomic number 6 and mass 12.00000

Atom 2 has atomic number 6 and mass 12.00000

Atom 3 has atomic number 6 and mass 12.00000

Atom 4 has atomic number 6 and mass 12.00000

Atom 5 has atomic number 6 and mass 12.00000

Atom 6 has atomic number 1 and mass 1.00783

Atom 7 has atomic number 1 and mass 1.00783

Atom 8 has atomic number 1 and mass 1.00783

Atom 9 has atomic number 1 and mass 1.00783

Atom 10 has atomic number 7 and mass 14.00307

Molecular mass: 78.03437 amu.

Principal axes and moments of inertia in atomic units:

1 2 3

EIGENVALUES -- 277.86089 314.48935 592.35024

X 0.98548 0.16979 0.00000

Y -0.16979 0.98548 0.00000

Z 0.00000 0.00000 1.00000

This molecule is an asymmetric top.

Rotational symmetry number 1.

Rotational temperatures (Kelvin) 0.31172 0.27541 0.14622

Rotational constants (GHZ): 6.49512 5.73864 3.04675

Zero-point vibrational energy 200961.1 (Joules/Mol)

48.03085 (Kcal/Mol)

Warning -- explicit consideration of 13 degrees of freedom as

vibrations may cause significant error

Vibrational temperatures: 574.10 630.23 856.82 971.39 1013.85

(Kelvin) 1155.83 1348.43 1384.74 1427.53 1457.38

1517.12 1547.78 1614.27 1754.68 1836.27

1959.15 2090.65 2130.16 2224.24 2319.73

4604.58 4621.32 4642.06 4657.76

Zero-point correction= 0.076542 (Hartree/Particle)

Thermal correction to Energy= 0.090828

Thermal correction to Enthalpy= 0.092642

Thermal correction to Gibbs Free Energy= 0.014561

Sum of electronic and zero-point Energies= -247.450049

Sum of electronic and thermal Energies= -247.435763

Sum of electronic and thermal Enthalpies= -247.433948

Sum of electronic and thermal Free Energies= -247.512029

E (Thermal) CV S

KCal/Mol Cal/Mol-Kelvin Cal/Mol-Kelvin

Total 56.995 29.537 85.509

Electronic 0.000 0.000 1.377

Translational 1.708 2.981 42.224

Rotational 1.708 2.981 27.399

Vibrational 53.579 23.575 14.508

**S2: Electron density maps and band gap energy profile for β-nicotyrine**

1. 2-D electron density contour map for β-nicotyrine

**
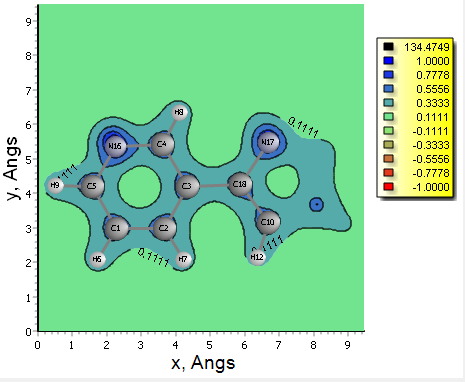
**

1. 3-D electron density map for β-nicotyrine


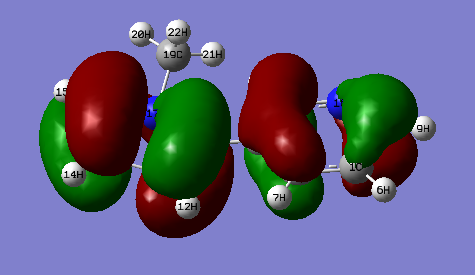


1. Molecular orbital profile for β-nicotyrine





**S3: Electron density maps and band gap energy profile for 3,5–dimethyl-1-phenylpyrazole**

1. 2-D electron density contour map for 3,5–dimethyl-1-phenylpyrazole


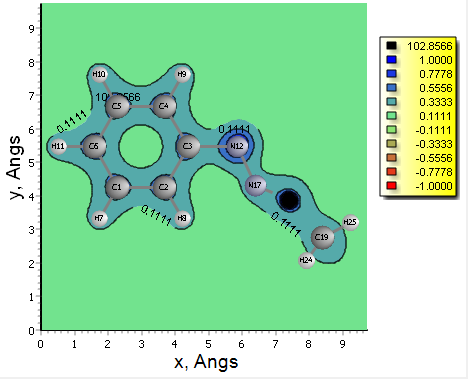


1. 3-D electron density contour map for 3,5-dimethyl-1-phenylpyarazole


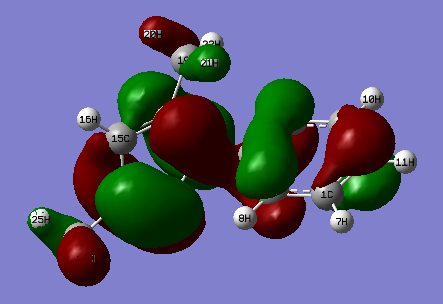


1. Molecular orbital diagram for 3,5-dimethyl-1-phenylpyrazole

##
